# Posterior-Only Sagittal Correction in Degenerative Lumbar Kyphosis Using Posterior Interpedicular Osteotomy (PIO) and Bilateral Discectomy with Hyperlordotic Cages: A Retrospective Case Series

**DOI:** 10.3390/jpm16090443

**Published:** 2026-08-24

**Authors:** Andrea Franchini, Giuseppe Rovere, Felice Barletta, Franco Lucio Gorgoglione, Giovanni Noia, Giuseppe Maccagnano, Andrea Perna

**Affiliations:** 1 Department of Orthopedics and Traumatology, Fondazione Casa Sollievo della Sofferenza IRCCS, 71013 San Giovanni Rotondo, Italy; a.franchini@operapadrepio.it (A.F.); f.barletta@operapadrepio.it (F.B.); f.gorgoglione@operapadrepio.it (F.L.G.);; 2 Department of Surgical Sciences, Policlinico Tor Vergata University Hospital, 00133 Rome, Italy; 3 Department of Clinical Sciences and Translational Medicine, Section of Orthopaedic and Traumatology, University of Rome Tor Vergata, 00133 Roma, Italy; 4 Orthopaedics Unit, Department of Clinical and Experimental Medicine, Faculty of Medicine and Surgery, University of Foggia, Policlinico Riuniti di Foggia, 71122 Foggia, Italy; giovanni.noia@unifg.it (G.N.); giuseppe.maccagnano@unifg.it (G.M.)

**Keywords:** lumbar kyphosis, posterior interpedicle osteotomy, TLIF, sagittal alignment, spinal fusion, degenerative spine, spinopelvic parameters

## Abstract

**Introduction**: Degenerative lumbar kyphosis (DLK) is a common cause of sagittal imbalance, pain, and functional disability. While three-column osteotomies provide powerful correction, they are associated with substantial morbidity. We evaluated the radiographic and clinical outcomes of a novel posterior-based correction strategy combining Posterior Interpedicular Osteotomy (PIO), bilateral discectomy, and hyperlordotic transforaminal lumbar interbody fusion (TLIF) in patients with DLK. **Materials and Methods**: A retrospective single-center case series was conducted including 215 consecutive patients with symptomatic DLK and pelvic incidence–lumbar lordosis (PI–LL) mismatch >10° treated between 2019 and 2023. All patients underwent PIO combined with bilateral discectomy and insertion of a 20° hyperlordotic TLIF cage. Radiographic parameters, including lumbar lordosis (LL), pelvic tilt (PT), and sacral slope (SS), were assessed preoperatively and up to 24 months postoperatively. Clinical outcomes were evaluated using the Visual Analog Scale (VAS), Oswestry Disability Index (ODI), and Short Form-36 (SF-36). Complications were systematically recorded. **Results**: Mean lumbar lordosis improved from 15.7° ± 8.3° preoperatively to 45.4° ± 11.6° at 24 months, corresponding to a mean correction of 29.7° ± 10.1° and 12.4° ± 2.9° per treated level (*p* < 0.001). Significant improvements were observed in PT, SS, VAS, ODI, and SF-36 scores (all *p* < 0.001), with maintenance of correction throughout follow-up. Mean operative time was 218.4 ± 47.3 min and mean blood loss was 483.2 ± 195.7 mL. The overall complication rate was 16.3%, with incidental durotomy being the most common event (14.9%). No cases of cage migration, deep infection, neurological deterioration, or radiographic pseudarthrosis were identified among patients who underwent CT evaluation. **Conclusions**: PIO combined with bilateral discectomy and hyperlordotic TLIF achieved substantial and durable sagittal correction with significant clinical improvement and an acceptable safety profile. By combining extensive posterior release with preservation of the anterior tension band, this technique may represent an effective intermediate alternative between conventional posterior column osteotomies and more invasive three-column deformity correction procedures.

## 1. Introduction

Degenerative lumbar kyphosis (DLK) is a progressive spinal disorder characterized by the loss of physiological lumbar lordosis secondary to advanced degenerative changes involving the intervertebral discs, vertebral endplates, and posterior spinal elements [1]. The resulting sagittal malalignment is associated with chronic low back pain, functional limitation, impaired quality of life, and progressive disability [2]. As compensatory mechanisms become exhausted, patients may develop fixed anterior sagittal imbalance with substantial deterioration in daily functioning [3]. The relationship between sagittal alignment and clinical outcomes has been extensively documented. Spinopelvic parameters, including lumbar lordosis (LL), pelvic incidence (PI), pelvic tilt (PT), and sacral slope (SS), are recognized as key determinants of spinal balance and postoperative outcomes. In particular, restoration of an appropriate pelvic incidence–lumbar lordosis (PI–LL) relationship has become a fundamental objective of deformity correction surgery [3,4]. Surgical treatment of DLK ranges from posterior column osteotomies, which provide limited angular correction, to more extensive three-column osteotomies such as pedicle subtraction osteotomy (PSO) and vertebral column resection (VCR), which offer greater corrective potential at the expense of increased operative complexity and morbidity [5]. Consequently, there remains a need for surgical strategies capable of achieving meaningful sagittal correction while maintaining an acceptable safety profile [5,6]. To address this challenge, we developed a novel posterior osteotomy technique, termed Posterior Interpedicular Osteotomy (PIO), designed to enhance segmental mobility and facilitate sagittal plane restoration when combined with bilateral discectomy and hyperlordotic transforaminal lumbar interbody fusion (TLIF). However, clinical evidence regarding the effectiveness and safety of this combined approach remains limited. The aim of the present study was to evaluate the radiographic correction, spinopelvic alignment, clinical outcomes, and complication profile associated with PIO combined with bilateral discectomy and hyperlordotic TLIF in a consecutive cohort of patients with degenerative lumbar kyphosis followed for 24 months.

## 2. Materials and Methods

### 2.1. Study Design and Ethical Considerations

This retrospective single-center case series was conducted and reported in accordance with the PROCESS (Preferred Reporting of Case Series in Surgery) guidelines [7]. The study was conducted in accordance with the principles of the Declaration of Helsinki and applicable national regulations. All procedures analyzed in this study were part of standard clinical care, and all patients provided written informed consent for surgical treatment and for the use of anonymized clinical and radiographic data for research purposes. In accordance with institutional policy for retrospective analyses of anonymized data derived from standard-of-care procedures, formal institutional review board approval was not required for this study. All patient data were fully de-identified prior to analysis to ensure confidentiality.

### 2.2. Patient Population

Consecutive patients who underwent PIO combined with bilateral discectomy and TLIF for DLK between January 2019 and December 2023 were screened for inclusion. Eligibility criteria included: (1) symptomatic DLK defined by a PI–LL mismatch greater than 10° on standing radiographs; (2) failure of at least 6 months of conservative treatment, including physical therapy, pharmacological management, and spinal injections when indicated; (3) minimum clinical and radiographic follow-up of 24 months; and (4) availability of complete preoperative and postoperative data. Exclusion criteria were: (1) traumatic or isthmic spondylolisthesis as the primary pathology; (2) spinal infection or neoplastic disease; (3) previous fusion surgery at the index levels; (4) severe untreated osteoporosis (T-score < −2.5); and (5) inability to complete patient-reported outcome measures.

A total of 215 patients met the inclusion criteria and were included in the final analysis. The cohort comprised 128 women (59.5%) and 87 men (40.5%), with a mean age of 63.4 ± 9.7 years and a mean body mass index of 27.3 ± 4.1 kg/m^2^. Baseline demographic and clinical characteristics are summarized in Table 1.

### 2.3. Surgical Technique

**Figure 1:**
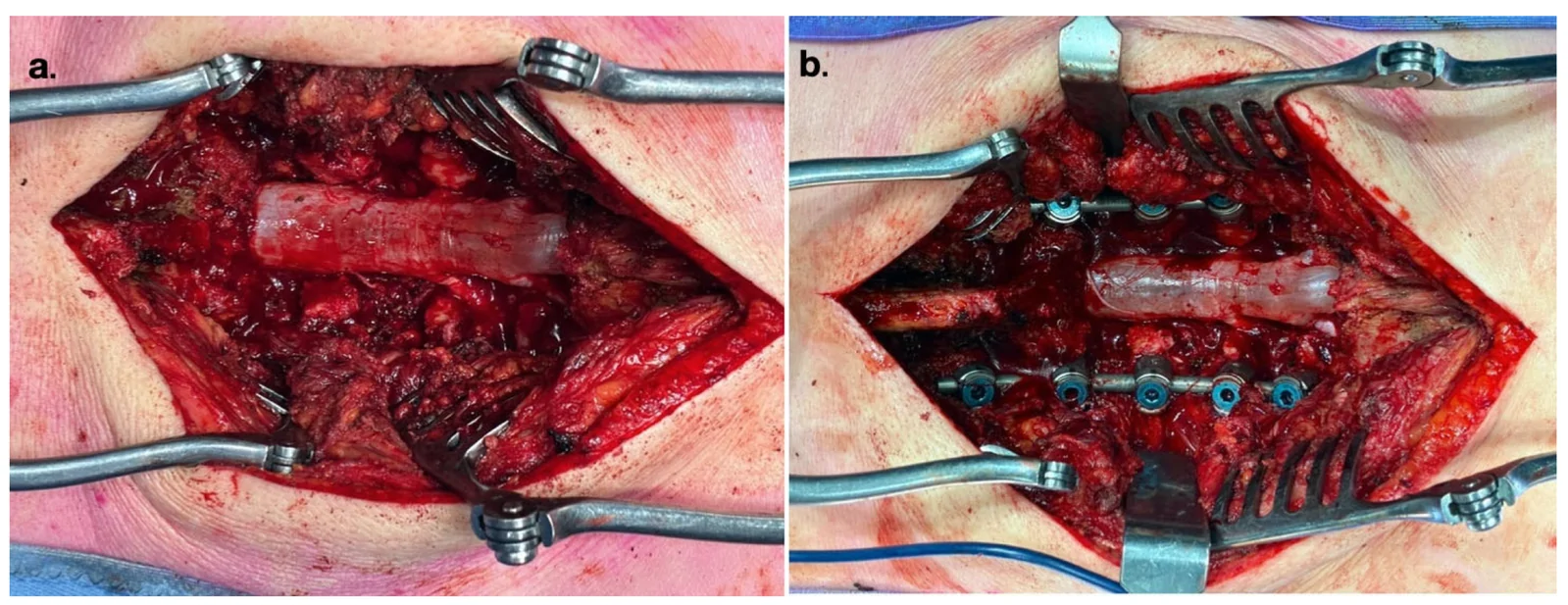
Intraoperative views of the surgical procedure. (**a**) Posterior interpedicular osteotomy (PIO) stage, showing the outlined contours of the L4 and L5 pedicles, with the dural sac and emerging nerve roots fully decompressed. (**b**) Final surgical field following bilateral pedicle screw-rod instrumentation and compressive tightening.

**Figure 2:**
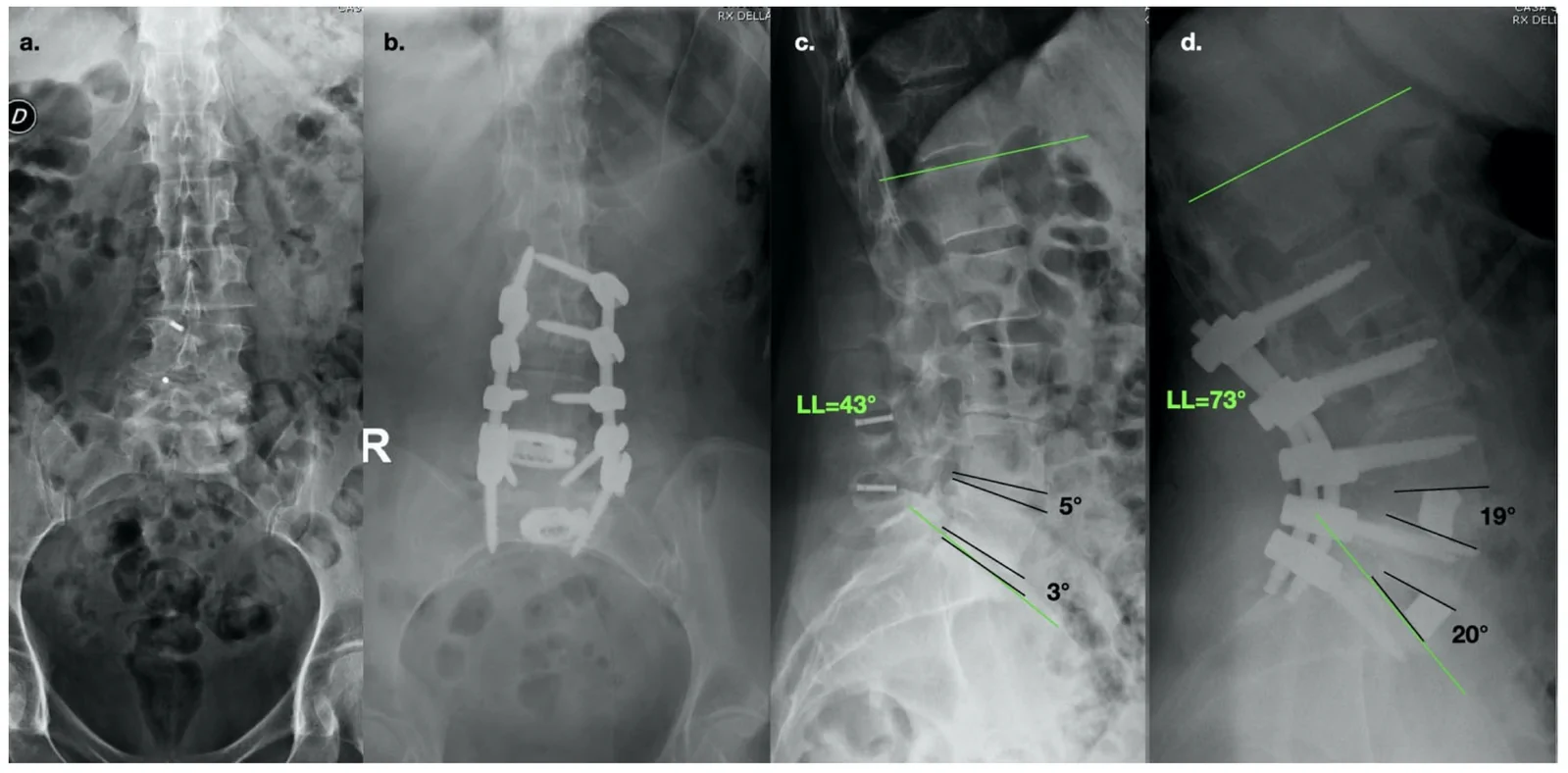
Radiographic evaluation of a 65-year-old female patient with prior implantation of two interspinous spacers, who underwent L2–S1 decompression and instrumentation, L4–L5 and L5–S1 posterior interpedicular osteotomies (PIO), and insertion of two hyperlordotic cages. (**a**) Preoperative anteroposterior (AP) and (**c**) lateral standing radiographs showing the previous interspinous devices. (**b**) Postoperative AP and (**d**) lateral standing radiographs at 24-month follow-up. A substantial regional and segmental correction is observed: global lumbar lordosis (LL) increased from 43° to 73°, with segmental lordosis improving from 5° to 19° at the L4–L5 level, and from 3° to 20° at the L5–S1 level.

All procedures were performed by the senior author using a standardized operative protocol. Patients were positioned prone on a radiolucent spinal table with the abdomen free and the hips maintained in extension to facilitate restoration of lumbar lordosis. Pedicle screw fixation was performed using the CD Horizon Solera system (Medtronic, Minneapolis, MN, USA). Following a standard posterior midline exposure, pedicle screws were inserted under fluoroscopic guidance. PIO was subsequently performed at each target level. Posterior Interpedicular Osteotomy (PIO) was conceived as an extended posterior release centered on the interpedicular corridor rather than as a conventional posterior column osteotomy. After pedicle screw placement, a bilateral en bloc resection of the osseoligamentous structures occupying the interpedicular space was performed. This included complete removal of the inferior articular process of the cranial vertebra, the superior articular process of the caudal vertebra, the ligamentum flavum, and the interlaminar bone, thereby creating a continuous bilateral interpedicular corridor. Unlike a standard Ponte osteotomy, whose primary objective is posterior column shortening through bilateral facetectomy, PIO is specifically designed to maximize segmental mobility by creating unrestricted bilateral access to the intervertebral disc. The pedicles, vertebral body, anterior annulus fibrosus, and anterior longitudinal ligament were intentionally preserved throughout the procedure. This anatomical configuration allows controlled posterior compression over hyperlordotic TLIF cages while maintaining the anterior tension band and avoiding disruption of the anterior column. Bilateral discectomy was then performed through the transforaminal corridors created by the osteotomy. Degenerated disc material was removed bilaterally and the vertebral endplates were prepared using standard curettes and rongeurs. Care was taken to preserve the anterior annulus fibrosus and anterior longitudinal ligament. A 20° hyperlordotic TLIF cage (L-ARTIC; Medtronic, Minneapolis, MN, USA) filled with locally harvested autograft and demineralized bone matrix was inserted into the anterior third of the disc space under fluoroscopic guidance. Following cage placement, pre-contoured rods were secured and segmental compression was applied to achieve sagittal correction. Final implant position and alignment were confirmed fluoroscopically prior to definitive fixation. Iliac fixation was added selectively in cases extending to L5–S1 with osteopenia or marked pelvic retroversion. Fusion constructs included L4–S1 in 107 patients (49.8%), L2–S1 in 55 patients (25.6%), and L2–L5 in 53 patients (24.7%). The mean number of instrumented levels was 2.8 ± 0.9. Intraoperative images are reported in Figure 1. An exemplificative case was reported in Figure 2.

### 2.4. Radiographic Assessment

Radiographic evaluation was performed using standardized standing full-length anteroposterior and lateral radiographs obtained preoperatively, within 48 h after surgery, and at 3, 6, 12, and 24 months postoperatively. Measurements were independently performed by two observers blinded to clinical outcomes using validated digital software (Surgimap Spine, version 2.3.2.1; Nemaris Inc., New York, NY, USA). The primary radiographic endpoint was LL, measured as the Cobb angle between the superior endplate of L1 and the superior endplate of S1. Secondary radiographic parameters included PI, PT, SS, and PI–LL mismatch. Segmental lordosis was evaluated exclusively at levels in which a TLIF cage was implanted. Measurements were performed using the Cobb angle between the inferior endplate of the cranial vertebra and the superior endplate of the caudal vertebra at each fused segment. Interobserver and intraobserver reliability were assessed using intraclass correlation coefficients (ICC).

### 2.5. Clinical Outcome Assessment

Clinical outcomes were assessed using validated patient-reported outcome measures, including the Visual Analog Scale (VAS) for back pain, the Oswestry Disability Index (ODI), and the Short Form-36 Health Survey (SF-36), including both Physical Component Summary (PCS) and Mental Component Summary (MCS) scores. Assessments were performed preoperatively and at 3, 6, 12, and 24 months postoperatively by personnel not involved in radiographic evaluation.

### 2.6. Complications

All intraoperative and postoperative adverse events occurring during the 24-month follow-up period were prospectively recorded and retrospectively reviewed. Complications were classified according to the Spine Adverse Events Severity System (SAVES -V2) [8] and categorized as intraoperative, early postoperative (<30 days), or late postoperative (>30 days). Revision procedures and their indications were analyzed separately.

### 2.7. Statistical Analysis

Statistical analyses were performed using IBM SPSS Statistics version 28.0 (IBM Corp., Armonk, NY, USA) and R version 4.3.1 (R Foundation for Statistical Computing, Vienna, Austria). Continuous variables are reported as mean ± standard deviation, whereas categorical variables are presented as frequencies and percentages. Data distribution was assessed using the Shapiro–Wilk test. Changes in radiographic and clinical outcome measures between preoperative and postoperative assessments were evaluated using paired *t*-tests or their nonparametric equivalents when appropriate. Longitudinal changes across follow-up time points were analyzed using repeated-measures analysis of variance with Greenhouse–Geisser correction when necessary, followed by Bonferroni-adjusted post hoc comparisons. Effect sizes were calculated using Cohen’s d. Interobserver and intraobserver reliability were assessed using intraclass correlation coefficients. Correlations between radiographic correction and clinical improvement at final follow-up were evaluated using Pearson correlation analysis. A two-sided *p* value < 0.05 was considered statistically significant.

## 3. Results

### 3.1. Patient Characteristics and Operative Data

Baseline demographic and operative characteristics are summarized in Table 1. Mean operative time was 218.4 ± 47.3 min (range, 140–390 min), with a mean estimated blood loss of 483.2 ± 195.7 mL (range, 120–1450 mL). Cell-salvage autotransfusion was used in 89 patients (41.4%), whereas 24 patients (11.2%) required allogeneic blood transfusion. Mean length of hospital stay was 6.8 ± 2.3 days. No procedure required intraoperative conversion to an alternative surgical strategy. Likewise, no intraoperative neurophysiological monitoring alerts resulted in modification or interruption of the planned procedure.

### 3.2. Radiographic Outcomes

Radiographic measurements demonstrated excellent reliability. Interobserver and intraobserver intraclass correlation coefficients for lumbar lordosis were 0.94 (95% CI, 0.91–0.96) and 0.97 (95% CI, 0.95–0.98), respectively. Similar reliability was observed for sacral slope and pelvic tilt measurements. Mean lumbar lordosis increased from 15.7° ± 8.3° preoperatively to 45.4° ± 11.6° at 24 months, corresponding to a mean correction of 29.7° ± 10.1° (*p* < 0.001). Mean segmental correction was 12.4° ± 2.9° per treated level. The mean number of treated levels with both PIO and hyperlordotic TLIF cage implantation was 2.0 ± 0.4 per patient. The overall increase in lumbar lordosis does not correspond to the simple arithmetic sum of the segmental corrections because global L1–S1 lordosis also reflects reciprocal changes occurring at untreated lumbar segments, rod contouring, and restoration of overall spinopelvic alignment following posterior instrumentation. No significant loss of correction was observed between the immediate postoperative evaluation and final follow-up (*p* = 0.312). Pelvic incidence remained unchanged throughout follow-up (54.8° ± 9.1° vs. 54.9° ± 9.3°; *p* = 0.78). Sacral slope increased significantly from 21.3° ± 7.5° to 36.9° ± 9.8° (*p* < 0.001), whereas pelvic tilt decreased from 28.6° ± 8.4° to 17.2° ± 6.9° (*p* < 0.001). Detailed radiographic outcomes are reported in Table 2.

### 3.3. Clinical Outcomes

Clinical outcome measures are summarized in Table 3. Mean VAS back pain scores improved from 7.4 ± 1.6 preoperatively to 2.0 ± 1.0 at 24 months (*p* < 0.001). VAS leg pain scores decreased from 6.1 ± 2.0 to 1.8 ± 0.9 during the same period (*p* < 0.001). Progressive improvement was observed across all postoperative follow-up intervals. Mean ODI improved from 58.4% ± 12.1% preoperatively to 22.7% ± 10.3% at 24 months (*p* < 0.001). SF-36 Physical Component Summary scores increased from 31.4 ± 7.8 to 48.9 ± 9.2, while Mental Component Summary scores improved from 38.7 ± 9.6 to 49.8 ± 10.3 (both *p* < 0.001). Clinical improvements achieved at 12 months remained stable through final follow-up, with no significant deterioration observed between the 12- and 24-month assessments. A significant positive correlation was identified between the magnitude of lumbar lordosis correction and improvement in ODI (r = 0.54, *p* < 0.001) as well as SF-36 PCS scores (r = 0.49, *p* < 0.001) at 24 months.

### 3.4. Complications

Thirty-five complications were recorded in 35 patients during the 24-month follow-up period, corresponding to an overall complication rate of 16.3%. Incidental durotomy was the most common adverse event and occurred in 32 patients (14.9%). In 31 cases, the dural defect was repaired intraoperatively without subsequent sequelae. One patient developed a symptomatic cerebrospinal fluid leak with pseudomeningocele formation, requiring revision surgery six weeks after the index procedure. Revision surgery included successful dural repair, resulting in complete resolution of the cerebrospinal fluid leak and pseudomeningocele. At the final follow-up, the patient had recovered uneventfully, with no persistent neurological deficits and no need for further revision surgery.

Two patients (0.9%) developed superficial wound infections that resolved with oral antibiotic therapy and local wound care. One patient (0.5%) demonstrated asymptomatic S1 pedicle screw breakage on routine follow-up imaging and was managed non-operatively because solid fusion and satisfactory clinical status were maintained.

No cases of deep infection, neurological deterioration, pulmonary embolism, cage migration, clinically significant subsidence, or new postoperative neurological deficit were observed.

Among the 186 of 215 patients (86.5% of the study cohort) who underwent CT evaluation at final follow-up, no radiographic evidence of pseudarthrosis was identified. Therefore, the absence of radiographic pseudarthrosis applies only to this subgroup of patients.

## 4. Discussion

### 4.1. Principal Findings

The present study evaluated the clinical and radiographic outcomes of PIO combined with bilateral discectomy and hyperlordotic TLIF in a consecutive cohort of patients with degenerative lumbar kyphosis. The principal findings were threefold. First, the technique achieved substantial and durable restoration of lumbar lordosis, with a mean correction of nearly 30° and an average segmental correction of 12.4° per treated level. Second, radiographic correction was accompanied by significant improvements in pain, disability, and health-related quality of life that were maintained throughout the 24-month follow-up period. Third, these outcomes were obtained with moderate blood loss, acceptable operative times, and a complication profile that compares favorably with more invasive deformity correction procedures.

### 4.2. Biomechanical Considerations

The degree of correction observed in this series is unlikely to be explained by the posterior osteotomy alone. Rather, it appears to result from the interaction of three complementary mechanisms: extensive posterior release through PIO, bilateral mobilization of the disc space, and anterior support provided by a hyperlordotic interbody cage [9]. A key feature of the technique is preservation of the anterior annulus fibrosus and ALL [10]. By maintaining the integrity of the anterior tension band while removing the principal posterior and middle-column restraints, posterior compression can be translated into controlled segmental lordosis rather than simple column shortening [11,12,13,14,15]. Under these conditions, the hyperlordotic cage functions as an anterior support point that facilitates angular correction and helps maintain the achieved alignment [13,14]. This concept may explain the relatively high segmental correction achieved in the present series compared with values typically reported for conventional TLIF procedures. Although the individual contribution of each component cannot be quantified from the current study design, the findings suggest that the combined construct provides greater corrective potential than would be expected from any single element alone [16].

### 4.3. Comparison with Existing Surgical Strategies

PIO may be viewed as an extended posterior release that occupies an intermediate position between conventional posterior column osteotomies and more aggressive three-column procedures [17,18]. Unlike a conventional Ponte osteotomy, which primarily increases flexibility through bilateral facetectomy and posterior ligamentous release, PIO specifically includes complete removal of the osseoligamentous structures occupying the interpedicular interval, thereby creating a wider bilateral interpedicular corridor. This extended exposure facilitates complete bilateral discectomy and insertion of hyperlordotic TLIF cages while preserving the pedicles, vertebral body, anterior annulus fibrosus, and anterior longitudinal ligament. Consequently, sagittal correction is achieved through the combined effect of extensive posterior release, bilateral disc mobilization, controlled posterior compression, and anterior support provided by the hyperlordotic cage rather than by posterior column shortening alone.

PIO should also be distinguished from the intradiscal osteotomy recently described by Carballo Cuello et al. [19]. Although both techniques aim to enhance sagittal correction while avoiding the morbidity of three-column osteotomies, their underlying surgical concepts differ. Intradiscal osteotomy primarily relies on intradiscal release and correction generated within the disc space itself. In contrast, PIO is based on enlargement of the posterior interpedicular corridor before disc preparation, facilitating complete bilateral discectomy and placement of hyperlordotic TLIF cages while preserving the anterior tension band. Therefore, correction results from the combined effect of extensive posterior release and anterior interbody support rather than from intradiscal osteotomy alone.

Compared with pedicle subtraction osteotomy [20], PIO preserves the pedicles, vertebral body, and anterior column, thereby avoiding vertebral wedge resection and potentially reducing blood loss, operative complexity, and neurological risk. Although direct comparisons cannot be made in the absence of a control group, the magnitude of correction achieved in the present series, together with the relatively low blood loss and acceptable complication profile, suggests that PIO may represent an effective intermediate option for sagittal realignment in selected patients. An additional practical advantage is that the entire procedure is performed through a single posterior approach without patient repositioning or disruption of the anterior longitudinal ligament [21] (Table 4).

### 4.4. Clinical Relevance of Spinopelvic Realignment

Correction of lumbar lordosis was accompanied by significant improvements in pelvic tilt and sacral slope, indicating restoration of a more physiological spinopelvic relationship. The reduction in pelvic retroversion is particularly relevant because pelvic compensation represents a finite mechanism used to maintain sagittal balance in the setting of lumbar hypolordosis. Furthermore, greater lordosis correction was associated with larger improvements in disability and physical health scores, supporting previous evidence that restoration of sagittal alignment is closely linked to functional recovery. These findings reinforce the importance of addressing global spinopelvic alignment rather than focusing exclusively on local decompression or segmental stabilization.

### 4.5. Complications

The most frequent complication was incidental durotomy, which occurred in 14.9% of patients. Although this rate is higher than that reported for routine lumbar fusion procedures, it is within the range described for complex deformity surgery and extensive posterior releases. The relatively high incidence of incidental durotomy may be partially explained by the extensive posterior release required to create the bilateral interpedicular corridor. Complete removal of the ligamentum flavum and the osseoligamentous structures occupying the interpedicular interval inevitably increases exposure of the dural sac and traversing nerve roots during bilateral discectomy. In our experience, this risk can be minimized by meticulous microsurgical dissection, progressive bilateral release performed under direct visualization, preservation of a clear tissue plane around the dura before complete ligamentum flavum removal, and careful handling of the neural structures during disc preparation and cage insertion. Importantly, nearly all cases were managed successfully with primary dural repair and did not result in clinically significant sequelae. Notably, no cases of deep infection, neurological deterioration, cage migration, or clinically significant subsidence were observed.

Among the 186 of 215 patients (86.5% of the study cohort) who underwent CT evaluation at final follow-up, no radiographic evidence of pseudarthrosis was identified. Therefore, the absence of radiographic pseudarthrosis applies only to this subgroup of patients. The remaining 29 patients did not undergo CT evaluation because our institutional follow-up protocol was primarily based on standardized standing radiographs, with CT reserved for cases requiring additional assessment of fusion status or implant integrity, thereby avoiding unnecessary radiation exposure.

While these findings should be interpreted cautiously because of the retrospective study design, they suggest that the technique provides satisfactory mechanical stability and favorable conditions for successful interbody fusion.

### 4.6. Strengths and Limitations

The strengths of this study include the relatively large consecutive cohort, the standardized surgical technique, the systematic radiographic assessment, and the comprehensive evaluation of patient-reported outcomes over a minimum follow-up of 24 months.

Several limitations should be acknowledged. The retrospective design introduces the possibility of selection bias and unmeasured confounding. The absence of a control group precludes direct comparison with alternative deformity correction strategies and limits attribution of the observed outcomes to any individual component of the procedure. Furthermore, the single-center, single-surgeon setting may limit the generalizability of our findings. Moreover, a learning curve is likely associated with the adoption of the PIO technique. Therefore, the operative time, estimated blood loss, and complication profile reported in the present series may reflect the experience of a high-volume spine surgeon and may not be immediately reproducible during the initial implementation of the technique in other centers. Finally, longer follow-up is required to determine the durability of correction and the incidence of adjacent segment degeneration or late mechanical complications. Future prospective comparative and multicenter studies are warranted to better define the role of PIO within the spectrum of surgical options for sagittal deformity correction and to evaluate its reproducibility across different surgical settings.

## 5. Conclusions

Posterior Interpedicular Osteotomy combined with bilateral discectomy and hyperlordotic TLIF provided substantial and durable correction of degenerative lumbar kyphosis, with significant improvements in sagittal alignment, pain, disability, and health-related quality of life at 24 months. The technique achieved a mean lumbar lordosis correction of 29.7° with an acceptable complication profile and without the morbidity typically associated with three-column osteotomies.

The corrective effect appears to derive from the combination of extensive posterior release, preservation of the anterior tension band, and anterior support provided by a hyperlordotic interbody cage. These findings suggest that PIO may represent a valuable intermediate option for patients requiring sagittal plane correction while avoiding the complexity of more aggressive osteotomy techniques.

## Figures and Tables

**Table 1:** Baseline demographic and operative characteristics (N = 215).

Variable	Value
Age, mean ± SD (years)	63.4 ± 9.7
Age range (years)	42–81
Female sex, n (%)	128 (59.5%)
Male sex, n (%)	87 (40.5%)
BMI, mean ± SD (kg/m^2^)	27.3 ± 4.1
ASA grade II, n (%)	121 (56.3%)
ASA grade III, n (%)	79 (36.7%)
Fusion construct L4–S1, n (%)	107 (49.8%)
Fusion construct L2–S1, n (%)	55 (25.6%)
Fusion construct L2–L5, n (%)	53 (24.7%)
Mean instrumented levels ± SD	2.8 ± 0.9
Mean cages implanted ± SD	2.0 ± 0.4
Mean operative duration (min) ± SD	218.4 ± 47.3
Estimated blood loss (mL) ± SD	483.2 ± 195.7
Mean hospital stay (days) ± SD	6.8 ± 2.3

Abbreviations: ASA, American Society of Anesthesiologists physical status classification; BMI, body mass index; SD, standard deviation.

**Table 2:** Radiographic outcomes: preoperative vs. 24-month postoperative values (N = 215).

Parameter	Preoperative Mean ± SD	24-Month Mean ± SD	Mean Change	*p*-Value	Cohen’s d
Lumbar Lordosis (LL, °)	15.7 ± 8.3	45.4 ± 11.6	+29.7 ± 10.1	<0.001	3.24
Segmental Correction/level (°)	—	—	12.4 ± 2.9	<0.001	—
Pelvic Incidence (PI, °)	54.8 ± 9.1	54.9 ± 9.3	+0.1 ± 1.1	0.78	—
Sacral Slope (SS, °)	21.3 ± 7.5	36.9 ± 9.8	+15.6 ± 6.2	<0.001	1.84
Pelvic Tilt (PT, °)	28.6 ± 8.4	17.2 ± 6.9	−11.4 ± 5.8	<0.001	1.73

**Table 3:** Clinical outcome scores across follow-up intervals (N = 215).

Outcome	Preop	3 Months	6 Months	12 Months	24 Months	*p*-Value
VAS Back (0–10)	7.4 ± 1.6	2.9 ± 1.4	2.4 ± 1.2	2.1 ± 1.1	2.0 ± 1.0	<0.001
VAS Leg (0–10)	6.1 ± 2.0	2.5 ± 1.3	2.1 ± 1.1	1.9 ± 1.0	1.8 ± 0.9	<0.001
ODI (%)	58.4 ± 12.1	32.1 ± 11.4	26.8 ± 10.9	23.5 ± 10.5	22.7 ± 10.3	<0.001
SF-36 PCS	31.4 ± 7.8	41.2 ± 8.7	45.3 ± 9.0	48.1 ± 9.1	48.9 ± 9.2	<0.001
SF-36 MCS	38.7 ± 9.6	43.4 ± 9.9	46.7 ± 10.1	49.0 ± 10.2	49.8 ± 10.3	<0.001

**Table 4:** Comparison of the main anatomical and surgical characteristics of posterior-based sagittal correction techniques.

Feature	Ponte Osteotomy	Intradiscal Osteotomy	Posterior Interpedicular Osteotomy (PIO)
Primary surgical target	Posterior column	Intervertebral disc	Interpedicular corridor
Bilateral facetectomy	Yes	Variable	Yes
Complete bilateral discectomy	No	Yes	Yes
Hyperlordotic TLIF cage	Optional	Optional	Standard component of the technique
Pedicles preserved	Yes	Usually	Yes
Anterior longitudinal ligament preserved	Yes	Variable	Yes
Main correction mechanism	Posterior column release	Intradiscal release	Posterior release + bilateral discectomy + hyperlordotic TLIF cage

Abbreviations: PIO, Posterior Interpedicular Osteotomy; TLIF, transforaminal lumbar interbody fusion.

## Data Availability

The original contributions presented in this study are included in the article. Further inquiries can be directed to the corresponding author.
